# Fusobacterium Nucleatum-Induced Tumor Mutation Burden Predicts Poor Survival of Gastric Cancer Patients

**DOI:** 10.3390/cancers15010269

**Published:** 2022-12-30

**Authors:** Yung-Yu Hsieh, Wen-Lin Kuo, Wan-Ting Hsu, Shui-Yi Tung, Chin Li

**Affiliations:** 1 Division of Gastroenterology and Hepatology, Department of Internal Medicine, Chang Gung Memorial Hospital, Chiayi 613016, Taiwan; 2 College of Medicine, Chang Gung University, Taoyuan 333323, Taiwan; 3 Department of Biomedical Sciences, National Chung Cheng University, Chiayi 621301, Taiwan

**Keywords:** *Helicobacter pylori*, *Fusobacterium nucleatum*, tumor mutation burden, gastric cancer

## Abstract

**Simple Summary:**

Gastric cancer is the fifth most common cancer in the world. An important risk factor in the development of alimentary tract cancers is the presence of pathogenic microbiota, such as *Helicobacter pylori*. We previously showed that sporadic infection of *Fusobacterium nucleatum* is associated with disease progression. Therefore, we examined the mutational landscape of *F. nucleatum*-positive, resected gastric cancer tissues using the Illumina TruSight Oncology 500 comprehensive panel to identify small nucleotide variants, small insertions and deletions, and unstable microsatellite sites. We identified a number of recurrent genetic aberrations, especially activating mutations of ERBB2, ERBB3, and PIK3CA and disrupted TP53. We found that the combination of *F. nucleatum* infection and high tumor mutational burden was a strong predictor of poor prognosis. Thus, *F. nucleatum* infection is correlated with increased accumulation of mutations and progression of gastric cancer, and these factors may be useful in the prognosis of this disease.

**Abstract:**

Co-infection of *Helicobacter pylori* and *Fusobacterium nucleatum* is a microbial biomarker for poor prognosis of gastric cancer patients. *Fusobacterium nucleatum* is associated with microsatellite instability and the accumulation of mutations in colorectal cancer. Here, we investigated the mutation landscape of *Fusobacterium nucleatum*-positive resected gastric cancer tissues using Illumina TruSight Oncology 500 comprehensive panel. Sequencing data were processed to identify the small nucleotide variants, small insertions and deletions, and unstable microsatellite sites. The bioinformatic algorithm also calculated copy number gains of preselected genes and tumor mutation burden. The recurrent genetic aberrations were identified in this study cohort. For gene amplification events, ERBB2, cell cycle regulators, and specific FGF ligands and receptors were the most frequently amplified genes. Pathogenic activation mutations of ERBB2, ERBB3, and PIK3CA, as well as loss-of-function of TP53, were identified in multiple patients. Furthermore, *Fusobacterium nucleatum* infection is positively correlated with a higher tumor mutation burden. Survival analysis showed that the combination of *Fusobacterium nucleatum* infection and high tumor mutation burden formed an extremely effective biomarker to predict poor prognosis. Our results indicated that the ERBB2-PIK3-AKT-mTOR pathway is frequently activated in gastric cancer and that *Fusobacterium nucleatum* and high mutation burden are strong biomarkers of poor prognosis for gastric cancer patients.

## 1. Introduction

Gastric cancer is the fifth most common cancer in the world. Besides the environmental and dietary factors, one important risk factor for alimentary tract cancers is pathogenic microbiota [1,2]. For the gastric epithelium, the cancer-induced bacterial pathogen is *Helicobacter pylori* [3]. It was estimated that 1–2% of *H. pylori*-infected patients eventually develop gastric cancer, prompting an effort to eradicate *H. pylori* infection to reduce the occurrence of gastric cancer [4]. In Taiwan, with decades of the *H. pylori* eradication program and routine upper gastrointestinal tract endoscope screening through the National Health Insurance, the incidence of gastric cancer indeed declined slowly [4]. However, according to the latest 2019 Cancer Registry Annual Report, gastric cancer is still the seventh most common cancer and the sixth in cancer-caused mortality in the male population. Furthermore, the 5-year survival rate is at about 40%, indicating the importance of improving the efficacy of current therapy.

*H. pylori* directly promotes the transformation of gastric epithelial cells. The type IV secretory machinery encoded by the cag pathogenicity island (cag PAI) allows the delivery of the virulent factor CagA and bacterial peptidoglycan to the gastric epithelial cells [5,6]. In the host cells, CagA modulates the function of the cytoskeleton and junctions and disrupts epithelium integrity [7,8,9]. Peptidoglycan, on the other hand, activates the PI3K-Akt signaling pathway to decrease apoptosis and promote cell growth [10,11]. In addition, *H. pylori* alters the microenvironment and incites a strong inflammatory response [9,12]. A change in the gastric microenvironment would allow further colonization of additional pathogenic microbes [3,13]. It is reasonable to speculate that these secondary infections could further promote cancer development and progression.

*Fusobacterium nucleatum* is a pathogenic bacteria found in oral microbiota. Recent studies demonstrated an enrichment of *F. nucleatum* in the colorectal cancer lesion-associated microbiota [14,15,16]. Increased attachment of *F. nucleatum* to the colorectal cancer cells is mediated through the interaction of bacterial lectin Fap2 with a tumor-specific Gal-GalNAc moiety on the cell surface [17]. Diagnostic detection of *F. nucleatum* in the stool sample could augment the fluorescence stool occult blood test and increase the detection sensitivity to more than 90% without compromising the specificity [18,19]. *F. nucleatum*, unlike *H. pylori*, possesses no molecular machinery to directly disrupt the intracellular signaling of the colorectal epithelium. However, *F. nucleatum* produces hydrogen sulfide [20,21], a secondary metabolite shown to exhibit DNA cytotoxicity [22,23]. This could be the underlying mechanism for the observation that *F. nucleatum* is specifically associated with high microsatellite instability of colorectal cancer [14,24]. Together, it appears that *Fusobacterium nucleatum* promotes oncogenesis by causing DNA damage. 

Our previous studies discovered that not only sporadic infection of *F. nucleatum* in gastritis patients, but also the abundance and frequency of *F. nucleatum* colonization, greatly increases the gastric cancer-associated microbiota [25]. The risk of *F. nucleatum* colonization progressively increases along with the disease progression, suggesting a tumor microenvironment continuously becoming more favorable for *F. nucleatum* colonization. In addition, gastric cancer patients with co-infection of *H. pylori* and *F. nucleatum* have poorer survival than those with only *H. pylori* infection or those without infection [26]. Since the patients included in our study all received gastrectomy, a poorer prognosis suggests that *F. nucleatum* increased aggressiveness and higher probability of metastasis of the gastric cancer cells. Thus, *F. nucleatum* could consecutively or synergistically collaborate with *H. pylori* to further promote gastric cancer progression. *F. nucleatum* produces hydrogen sulfide, a metabolite causing cell DNA damage and potentially increasing the mutations of the cancer cells. In this report, we provide evidence demonstrating that *F. nucleatum* infection increases the tumor mutation burden of gastric cancer and greatly shortens patients’ survival time. Our findings suggest that *F. nucleatum* plays a pivotal role in aggravating the progression of gastric cancer by accelerating the accumulation of mutations in cancer cells.

## 2. Materials and Methods

### 2.1. Study Cohort and Specimens

This study was approved by the Institutional Review Board of Chiayi Chang Gung Memorial Hospital (Institutional Review Board, approval No.202001246B0). Acquisition and use of clinical specimens were carried out in accordance with the Declaration of Helsinki. Frozen resected cancer tissues were obtained from Chiayi Chang Gung Memorial Hospital Tissue Bank. The status of *H. pylori* infection was determined by a standard rapid urease test at the time of specimen collection. In all, 36 resected gastric cancer tissues were analyzed. The frozen tissues were pulverized in TRI reagent (Thermo Fisher Scientific, Waltham, MA, USA) and centrifuged to remove undissolved debris. Total DNA, including both cellular and microbial DNA, was extracted according to the manufacturer’s protocol, and the concentration of DNA was determined by Qubit dsDNA high-sensitivity fluorometric quantification assay (Thermo-Fisher). The presence of *F. nucleatum* in the specimens was determined by nested PCR detection of the NusG gene, as described previously [26].

### 2.2. Mutation Analysis

DNA mutation profile was analyzed using TruSight Oncology 500 panel (Illumina, San Diego, CA, USA) [27,28]. This targeted panel interrogates the exon sequences of 523 preselected cancer-associated genes for mutation analysis. In addition, the panel interrogates 125 microsatellite sites to estimate the microsatellite instability status. Copy number gains for the selected 57 genes and tumor mutation burden were calculated from the sequencing reads through bioinformatic analysis. 

To perform the analysis, the specimen DNA was first enzymatically fragmented using dsDNA fragmentase (NEB) and purified using magnetic size selection matrix (Agencourt AMPure XP, Beckman Coulter, Brea, CA, USA). The average size and concentration of fragmented DNA were analyzed by capillary electrophoresis using the D1000 ScreenTape assay on a TapeStaton 2200 analyzer (Agilent, Santa Clara, CA, USA) and fluorescence quantification (Qubit). Fragmented DNA was subsequently used as the input material for TruSight Oncology 500 panel library construction. The manufacturer’s protocol was followed closely. Briefly, fragmented DNA was first repaired to blunt-ended DNA. A single A residue was then attached to the 3′-end of the blunt-ended fragments. In the next step, the adaptor with unique molecular index (UMI) was ligated to the A-tailed DNA fragments. Adapted-ligated fragments were purified using a magnetic size selection matrix, followed by PCR amplification with Illumina i5 and i7 primers carrying index sequences. The library was then mixed with biotin-labeled capture oligomers in the hybridization reaction on a PCR cycler for 18 to 24 h. Target sequences were then captured using streptavidin magnetic beads. The beads were then extensively washed to remove non-specifically bound DNA. After washing, captured DNA fragments were released from the beads under alkaline conditions, and the released DNA’s pH was neutralized by adding pH neutralizing buffer. The enriched library was then subjected to a second round of biotin-labeled oligomer hybridization for 4 h. Target sequence capture was carried out again using streptavidin magnetic beads. After the second round of capture, the streptavidin in beads was washed once using low salt buffer, and the recaptured library was eluted by alkaline buffer and neutralized by pH neutralizing buffer. The enriched library was then amplified to yield the final sequencing-ready library. The sequencing-ready library was then analyzed by capillary electrophoresis and fluorescence quantification, as described above.

Sequencing was carried out on an Illumina NovaSeq 6000 sequencer for 300 paired-end cycles. The resulting forward and reverse reads were trimmed to 100 bp as specified by the manufacturer for subsequent bioinformatic analysis. Analysis was carried out using a TSO500 local app 2.0.0.70 on a local workstation. The analysis pipeline first mapped the sequencing reads to human genome hg19, followed by UMI collapsing and remapping to hg19 to produce the BAM files. The BAM files were annotated to create the VCF files. The VCF file was uploaded to Illumina BaseSpace Variant Interpreter for variant visualization and classification analysis. The status of microsatellite instability was represented as a percentage of the unstable sites among detected microsatellite sites. The variants identified through the assay were then filtered against GnomAD Exome, GnomAD Genome, and 1000 genomes database to remove common and frequent variants in populations. The remaining variants were then used to calculate tumor mutation burden, which was represented as the number of somatic mutations per Mb.

### 2.3. Statistical Analysis

Statistical analysis of tumor mutation burden between *F. nucleatum*-negative and positive patients was performed by Student’s *t*-test. Survival probability was calculated with Kaplan–Meier analysis.

## 3. Results

Our previous study demonstrated that co-infection of *F. nucleatum* and *H. pylori* predicts poorer survival for gastric cancer patients. *F. nucleatum* was shown to produce cytotoxic metabolites, such as H2S, and was associated with microsatellite instability-high colorectal cancer, suggesting that *F. nucleatum* may cause hypermutation of the cancer genome. We hypothesized that *F. nucleatum* promotes gastric cancer progression through a similar molecular mechanism. An increase of mutations in the genome can be represented by higher tumor mutation burden. To validate this hypothesis, we hence compared the mutation landscape of resected gastric cancer tissues with co-infection of *H. pylori* and *F. nucleatum* and those with only *H. pylori* infection. The TSO500 DNA panel queries the exon sequences of preselected 523 genes and 125 microsatellite sites. The sequencing reads were analyzed by the TSO500 local app provided by the manufacturer to identify variant identification and unstable microsatellite sites. Using the sequencing data, the analysis package calculated tumor mutation burden (TMB) and microsatellite instability (MSI) as the percentage of unstable sites. The analysis pipeline also calculates the potential copy number gains of 57 genes in the panel. The TMB obtained through the TSO500 analysis pipeline is shown to be comparable to other FDA-approved clinical diagnostic services, including Foundation One CDx. Hence, we selected the TruSight Oncology 500 comprehensive cancer panel (TSO500) for achieving the experimental goal of assessing the mutation landscape of the resected cancer specimens.

**Figure 1:**
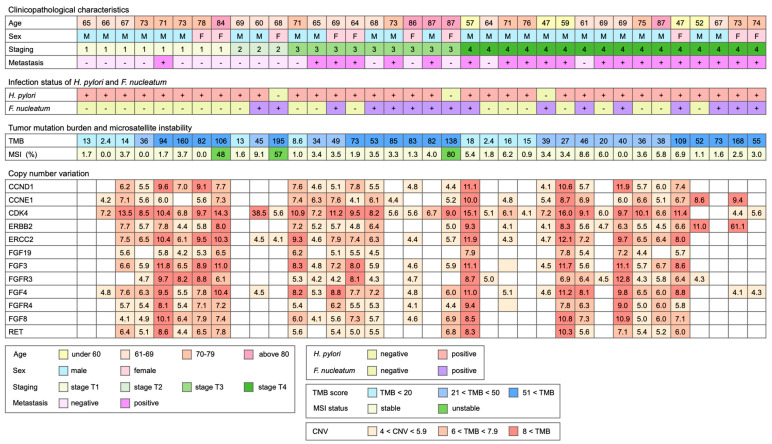
The clinical and pathological characteristics of the patients in this study. Resected cancer tissues from gastrectomy were analyzed using TruSight Oncology 500 DNA panel. Tumor mutation burden, microsatellite instability, and copy number variation calculated by TSO500 local app are listed. TMB represents the number of somatic variants per megabase of targeted region, and micro-satellite represents the percentage of unstable sites over all identified microsatellite sites. Copy number variation represents the copy number calculated by the analysis algorithm.

Following our previous investigation, we focused on the subset of *H. pylori*-positive gastric cancer patients with or without *F. nucleatum* infection. In this study, 36 resected gastric cancer tissue specimens, including twenty specimens with *H. pylori* infection, thirteen specimens with co-infection of *H. pylori* and *F. nucleatum*, and three specimens with only *F. nucleatum* infection, were analyzed using a TSO500 DNA panel. The age, gender, staging, metastasis, and status of *H. pylori* and *F. nucleatum* infection for the analyzed specimens are shown in Figure 1. Stratified by cancer stage, eleven specimens were from the patients of early-stage (stages 1 and 2), while twenty-five specimens were from the patients of late-stage (stages 3 and 4) disease. In the early-stage group, there were two specimens positive for *F. nucleatum*, while fourteen specimens were positive for *F. nucleatum* infection in the late-stage group. This is similar to our previous observation that patients with late-stage gastric cancer are more prone to *F. nucleatum* infection. The two biomarkers for assessing the overall genomic mutation load, TMB and MSI, are also shown in Figure 1. However, MSI assessment indicated that only three specimens were considered MSI-high (percentage > 20%). Although all three patients are female patients, it is likely a coincidence. Other than that, there is currently no clear association of the MSI-high status with other clinicopathological characteristics of the patients.

In contrast to MSI, TMB in the cohort ranged between 2.4 and 195, with the median at 45.5. We hence arbitrarily stratified the specimens into three groups based on the number of TMB. Nine specimens with TMB smaller than 20 were considered as low TMB, eleven specimens with TMB between 20 and 50 were considered as medium TMB, and sixteen specimens with TMB larger than 50 were considered as high TMB. Using this arbitrary cut-off to stratify the specimens, there were four low TMB (36.4%), two medium (18.2%), and five high TMB (45.5%) specimens in the early-stage group. On the other hand, there were five low TMB (20%), nine medium TMB (36%), and eleven high TMB (44%) specimens in the late-stage patients. It appears that a similar percentage of patients have high TMB in both early-stage and late-stage groups, hence indicating that high TMB is not associated with cancer staging.

In addition to TMB and MSI, copy number variation was also reported by the TSO500 local app analysis package. Frequent amplification events are shown in Figure 1. Among these gene copy number gain events, amplification of ERBB2 is the indication for the anti-HER2 treatment [29]. In our cohort, the amplification event of ERBB2 determined by TSO500 was found in twenty-four (66.7%) specimens, much higher than the reported 6% prevalence of HER2-positive gastric cancer in Taiwan. The standard diagnostic methods for HER2-positive gastric cancer are immunohistochemistry staining and fluorescence in situ hybridization. This discrepancy in the prevalence of HER2 amplification, hence, could arise from the different sensitivities of detection methods. Alternatively, it is also possible that the protein level is not directly associated with the copy number of ERBB2. 

Besides the amplification of ERBB2, we also observed that CCND1, CCNE1, and CDK4 were also frequently amplified. Amplification of these cell cycle regulators likely pushes cell cycle progression and promotes cancer growth. Particularly, CDK4 was found to be amplified in nearly all specimens we examined (88.9%). CDK4 is also a therapeutic target, although anti-CDK4/6 therapy is currently indicated for metastatic breast cancer [30,31]. Whether anti-CDK4/6 therapy provides clinical benefits to gastric cancer patients remains to be determined. Other frequently amplified genes are members of the fibroblast growth factors and the receptors. Here, we identified the amplification of FGFR3 and FGFR4. The oncogenic role of the FGFs and FGFRs in gastric cancer have been previously demonstrated [32]. Anti-FGFR3 activation mutation therapy is approved for the treatment of bladder cancer carrying FGFR3 activation mutation, but we did not observe activation mutation of FGFR3 in these specimens. Hence, whether FGFR amplification in gastric cancer could be a therapeutic target remains to be investigated.

**Figure 2:**
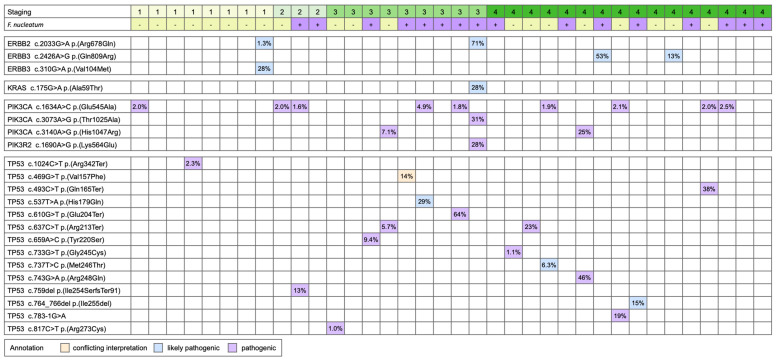
The pathological and likely pathogenic variants identified in the study cohort.

All variants with variant allele frequency (VAF) larger than 1% were analyzed for recurrent features. We first focused on those variants classified as likely pathogenic and pathogenic in the ClinVar database. Recurrent variants are listed in Figure 2. The analysis showed that the activation mutation of ERBB2 p.(Arg678Gln) was identified in stage-1 and stage-3 specimens that have ERBB2 copy numbers of 5.8 and 5, respectively. Additional pathogenic mutations, including KRAS p.(Ala59Thr), PIK3CA p.(Thr1025Ala), and PIK3R2 p.(Lys564Glu), were identified in that particular stage-3 specimen, suggesting that the tumor is refractory to the anti-HER2 treatment [33,34,35]. On the other hand, the stage-1 specimen carries an additional ERBB3 p.(Val104Met) mutation. In addition to this specimen, two other stage-4 specimens carry the ERBB3 p.(Gln809Arg) mutation. HER3 is known to form a heterodimer with HER2 and may also contribute to treatment resistance [36,37], although the precise role of ERBB3 in gastric cancer remains to be investigated. 

In addition to ERBB2 and ERBB3 mutations, PIK3CA was also frequently activated in our study cohort. Amongst the specimens with PIK3CA p.(Glu545Ala) mutation, one with PIK3CA p.(Thr1025Ala) and two with PIK3CA p.(His1047Arg) were identified. The specimens with activated PIK3CA accounted for one-third of analyzed specimens. The PIK3CA activation mutations are the indication for the PIK3CA inhibitor regimen for advanced or metastatic breast cancer, but their efficacy on PIK3CA-mutated gastric cancer is still under investigation. Besides cancer-driving activation mutations, frequent loss-of-function of TP53 was identified in our cohort as well. In fifteen specimens, there were eleven pathogenic, three likely pathogenic, and one VUS (variant of uncertain significance; conflicting interpretation), TP53 mutations. Activated PIK3CA mutations and loss-of-function of TP53 were concurrently found in seven specimens. These mutations appeared to not be associated with the status of *F. nucleatum* infection since approximately equal numbers of *F. nucleatum*-positive and *F. nucleatum*-negative specimens carry these mutations.

**Figure 3:**
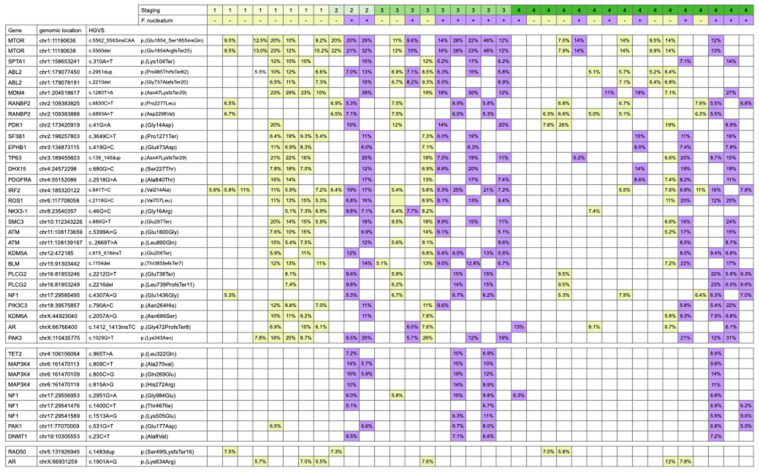
The variants in the SNV/INDEL identified in the specimens share common features.

The TSO500 local app analyzed the variants in the SNV/INDEL VCF file and filtered against the population database, including GnomAD and 1000 Genomes, to produce the list of variants used to calculate TMB. We next investigated whether those variants identified in the specimens have common features. The analysis result is shown in Figure 3. The finding demonstrates that many recurrent mutations were found in multiple specimens, regardless of the status of *F. nucleatum* infection. Among these mutations, the missense mutations and the frameshifting mutations are expected to produce novel protein sequences and could be processed and presented to the adapted immune system. Hence, the variants identified here could be further examined for their potential to stimulate an anti-tumor immune response.

**Figure 4:**
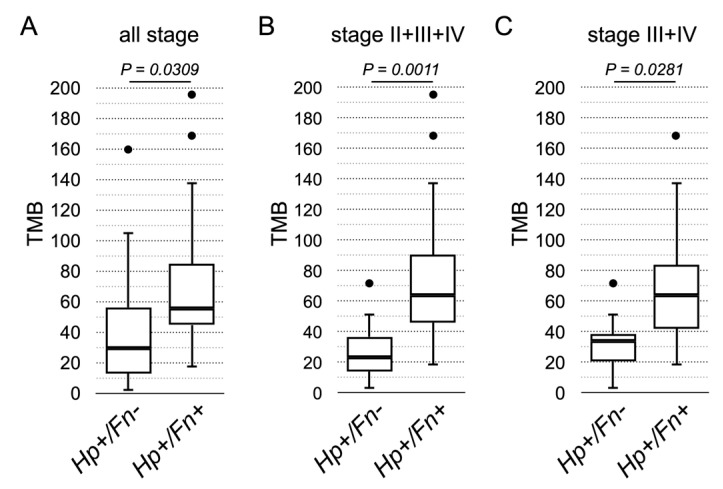
The statistical analysis of TMB between the *F. nucleatum*-positive and *F. nucleatum*-negative specimens. (**A**) All specimens of all stages were included for analysis (Fn− group n = 20, Fn+ group n = 16). (**B**) Specimens of stages 2 to 4 were included for analysis (Fn− group n = 12, Fn+ group n = 13). (**C**) Specimens of stages 3 and 4 were included for analysis (Fn− group n = 9, Fn+ group n = 10).

The hypothesis that *F. nucleatum* increases mutation burden was then examined using the TMB reported by the TSO500 local app. Analysis was performed to examine the TMB between the *F. nucleatum*-positive and *F. nucleatum*-negative specimens. The result of statistical significance was achieved when all specimens of all stages were included for analysis (*p* = 0.0309), specimens of stages 2 to 4 (*p* = 0.0011), or specimens of stages 3 and 4 (*p* = 0.0281) were included for analysis (Figure 4). The analysis clearly demonstrated that the *F. nucleatum*-positive specimens indeed have higher TMB than the *F. nucleatum*-negative specimens.

**Figure 5:**
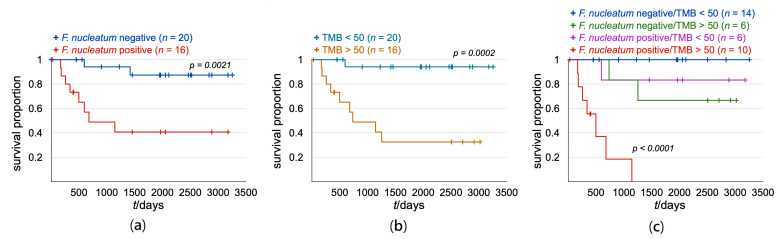
The Kaplan–Meier analysis of the patients. (**a**) The analysis carried out by stratifying the patients into the *F. nucleatum*-negative and -positive groups. (**b**) The analysis carried out by stratifying the patients into the TMB < 50 and TMB > 50 groups. (**c**) The patients were striated into four groups for survival analysis according to the status of *F. nucleatum* and TMB. The statistical significance was obtained between the patient without *F. nucleatum* infection and TMB <50 and those with *F. nucleatum* infection and TMB > 50.

We then analyzed the association between TMB, *F. nucleatum* infection, and patients’ survival. When the specimens were stratified into two groups using the status of *F. nucleatum* infection, the survival was significantly worse in the group positive with *F. nucleatum* infection (Figure 5a). The result is consistent with our previous study that *F. nucleatum* is a poor survival factor. The median TMB of all specimens in this study cohort was 45.5. Hence, we used TMB 50 as the stratification criteria to stratify the patients. The survival analysis showed that the prognosis of the patients with TMB > 50 was significantly poorer than in patients with lower TMB (Figure 5b). The status of *F. nucleatum* infection and TMB were further combined to stratify the patients into four groups for analysis. The results showed that patients without *F. nucleatum* infection and TMB < 50 could expect an excellent prognosis (Figure 5c). On the other hand, *F. nucleatum* infection and TMB > 50 each exerted a slight impact on patient survival, but the number of specimens was not large enough to reach a statistical and conclusive result. Strikingly, the survival curve of the patients positive for *F. nucleatum* infection and TMB > 50 decreased precipitously. Hence, our results clearly show that the combination of *F. nucleatum* and TMB served as an effective biomarker for gastric cancer prognosis.

## 4. Discussion

The interaction between microbiota and host cells plays a pivotal role in cancer development and progression. Besides *H. pylori*, *F. nucleatum* has been demonstrated to be associated with poor clinical prognosis of gastric cancer. In this report, we further showed that *F. nucleatum* promotes the accumulation of tumor mutations in gastric cancer. Analysis of our study cohort using a TSO500 comprehensive panel and the analysis algorithm indicated that TMB 50 could serve as an effective cut-off to predict the survival of the patients. In addition, TMB augments the survival prediction power of *F. nucleatum*, and these two factors together form a compound biomarker for poor prognosis with extremely effective prediction power. However, the size and population characteristics of the study cohort were limited, so it is unclear whether similar observations can be made in other patient populations. In addition, the findings are correlational, and the underlying mechanism remains to be illuminated. Our studies, nevertheless, provide observational evidence to suggest how *F. nucleatum* plays a role in gastric cancer. First, the patients infected with *H. pylori* have a higher risk of secondary *F. nucleatum* infection, likely due to the alternation of a gastric environment that favors *F. nucleatum* colonization. According to our study, only three patients were infected with *F. nucleatum* in the absence of *H. pylori* infection, and approximately one-third of the thirty-three *H. pylori*-positive patients were infected with *F. nucleatum*. In the mouse model, *F. nucleatum* was unable to consistently colonize the gastric epithelium [38], supporting the conjecture that pre-colonization of *H. pylori* could be a precondition for *F. nucleatum* colonization.

Mutation profiling shows a mismatch between clinical pathogenic mutations with the *F. nucleatum* infection status. This finding suggests that *F. nucleatum* does not promote cancer aggressiveness by inducing specific cancer-driving mutations. In contrast to the incidental arising of specific cancer-driving mutations, the analysis reveals a strong correlation between *F. nucleatum* and high TMB (>50). The survival analysis clearly indicates that high TMB predicts poor prognosis, suggesting that high TMB is associated with increased aggressiveness. This pathogenic effect could be mediated through the release of genotoxic hydrogen sulfide. On the other hand, it could be possible that *F. nucleatum* exerts additional pathogenic effects to further increase the aggressiveness and, likely, the metastasis potential of gastric cancer. One such mechanism is through the suppression of the anti-tumor response [16,39]. *F. nucleatum* has been shown to modulate the immune response in colorectal cancer [40] and is correlated with a better response of immune checkpoint inhibitors through the upregulation of PD-L1 in cancer cells [41]. Hence, *F. nucleatum* infection could alter the tumor microenvironment in a way that allows rapid evolution and escape of the cancer cells. 

For gastric cancer treatment, the majority of patients would receive gastrectomy to remove the lesion. However, the recurrence rate is high, and the prognosis of gastric cancer remains relatively poor. Anti-HER2 treatment is the standard treatment regimen for those HER2-positive gastric cancer patients [29]. Our analysis showed that ERBB2 and ERBB3 were either amplified or activated in a significant portion of specimens. Furthermore, one of the downstream pathways of HER2 is the PI3K-AKT-mTOR pathway. Our finding that the PIK3CA activation mutation is found in a relatively high percentage of specimens suggests that the PI3K-AKT-mTOR pathway may be frequently activated to bypass HER2 activation [33]. Together, activation of the HER2 signaling could be one of the common oncogenic mechanisms in gastric cancer. If so, restoring the sensitivity by targeting activated HER2 or its downstream pathway should provide clinical benefits to those HER2 treatment-refractory patients. 

In addition to targeted therapy, immune checkpoint inhibitor immunotherapy was approved by FDA to treat advanced gastric cancer. In 2019, immunotherapy for gastric cancer was included in the reimbursement plan of Taiwan National Health Insurance. However, since the overall response rate for liver and gastric cancers was less than 20%, the reimbursement plan was revised to remove liver and gastric cancers from the reimbursement plan in just one year. It is likely that the low response rate of immunotherapy was due to the lack of effective inclusion criteria. Hence, it is essential to search for a more effective predictive biomarker for the immunotherapy of gastric cancer. TMB has been investigated as a predictive biomarker for clinical response to immune checkpoint inhibitor immunotherapy. High TMB is associated with clinical response in melanoma and lung cancers, but the evidence for the usefulness of TMB in guiding the immunotherapy of gastric cancer was promising but remained inconclusive [42,43,44]. It was shown that the gut microbiota is associated with the response and toxicity of immune checkpoint inhibitor treatment. Similarly, the gastric cancer-associated microbiota may also play a role in response to immunotherapy [45,46,47].

## 5. Conclusions

Future investigation is required to examine whether *F. nucleatum* could augment TMB to more effectively identify the patients who will benefit from immunotherapy. On the other hand, eradication of *F. nucleatum* could provide clinical benefits and meaningfully extend the survival of the patients.

## Data Availability

The sequencing data are deposited in Sequence Read Archive, National Center for Biotechnology Information, USA. The BioProject accession number is PRJNA841034.
